# Health behaviors, health, sociodemographic factors, and school success in adolescence as risk factors for injury deaths: a longitudinal study

**DOI:** 10.1186/s12889-025-23214-0

**Published:** 2025-05-29

**Authors:** Alisa Teuho, Ville Ponkilainen, Leena Koivusilta, Arja Rimpelä, Ville M. Mattila

**Affiliations:** 1 Faculty of Medicine and Life Sciences, University of Tampere, Tampere, Finland; 2 Department of Orthopedics and Traumatology, Tampere University Hospital, Tampere, Finland; 3 Department of Social Research, Faculty of Social Sciences, University of Turku, Turku, Finland; 4 Unit of Health Sciences, Faculty of Social Sciences, Tampere University, Tampere, Finland; 5 Department of Adolescent Psychiatry, Tampere University Hospital, Tampere, Finland

**Keywords:** Health, Health behaviors, Adolescence, Injury death, Traumatology, Epidemiology

## Abstract

**Background:**

Injuries are a substantial cause of mortality in young adults. Previous longitudinal studies on the impact of adolescent health behaviors, health, sociodemographic factors, and school success on injury deaths are lacking. We examined the influence of these factors in adolescence on later injury death.

**Methods:**

We conducted a population-based longitudinal study with an average 26-year follow-up, using questionnaire data from the Adolescent Health and Lifestyle Survey (AHLS) and register data. Adolescents aged 14, 16, or 18 years who answered the survey between 1981 and 1997 were included. A total of 47 326 individuals responded to the survey. Causes of death were obtained from the Finnish official Cause-of-Death Register. Cox regression model was used to analyze the associations between explanatory variables and injury death. Adjusted hazard rations (aHR) and 95% confidence intervals (CI) were computed. 14-year-olds and 16–18-year-olds were analyzed separately by sex.

**Results:**

We identified 550 injury deaths, 432 in men and 118 in women. The mean age at death was 30 years. Drinking style (recurring drunkenness 14-year-old girls aHR 4.35 CI 1.00–19.02 and boys aHR 4.02, CI 1.62–10.00; 16–18-year-old girls aHR 2.63, CI 1.13–6.13 and boys aHR 1.70, CI 1.07–2.71) was associated with injury death in all subgroups. Smoking (girls aHR 2.00, CI 1.21–3.33 and boys aHR 1.86, CI 1.42–2.44) and stress symptoms (two or more/day girls aHR 2.34, CI 1.32–4.14 and boys aHR 2.07, CI1.39–3.07) were associated with injury death in 16–18-year-olds. Not living with both parents also increased the risk of injury death in boys.

**Conclusion:**

Our findings suggest that adolescents who drift into risky health behavior and struggle with stress symptoms are at higher risk for injury death later in life. More support should, therefore, be allocated to these groups during adolescence.

**Supplementary Information:**

The online version contains supplementary material available at 10.1186/s12889-025-23214-0.

## Background

On average, injury deaths account for 8% of annual deaths worldwide [1]. The proportion is even more substantial among young people [1–3]. In Finland, injuries accounted for 9% and suicides for 7% of all deaths among the working-age population in 2020 [4]. Globally, the number of injury deaths increased by 2.3% between 2007 and 2017, with the greatest increase in self-harm and interpersonal violence [5]. An increase in injury deaths has also been observed between the ages of 15 to 20 years, especially among men [3, 5, 6] who are known to be at higher risk for death from unnatural causes [7, 8].

Regarding the risk of injuries, adolescence is an important phase of life. Risky health behaviors like alcohol use are adopted at teenage; the use of alcohol and subsequent intoxication are known to increase the risk of injury death [9]. Adolescent health behavior can predict risk-taking behavior and health in later life, too [5]. In adolescence, decisions on the selection of a school track and the continuation of schooling after compulsory education are to be made, often based on previous school success. These predict education level and socioeconomic status (SES) in adulthood [10, 11], which in turn increase the risk of injury death among adults [12].

Socioeconomic circumstances of the childhood family shape both the adoption of risky behaviors as well as the school career of the adolescent, which in later life will have a wide-ranging effect on an individual´s health [13, 14]. Adolescents from low SES families are more prone to adopt health compromising and risk-taking behaviors [15], whereas among adolescents from families with a higher SES healthy behaviors are more common, e.g., frequent participation in organized sports [16]. In addition, low family SES and family stressors are associated with mental health problems [17, 18] and poor perceived health [19]. Social disadvantage and stressful life events in childhood increase the risk for injury death among adolescents and young adults [7], and injury deaths are more common among children whose parents have a low education [13, 20–23], manual occupational status, low family income [13, 24], and who are not living in a two-parent family [25]. Further, injury deaths are more common among children living in rural or deprived areas [3, 12].

Parents’ education and SES shape a child’s school success [26]. Children from low SES families do worse at school than their classmates from families of higher SES. Poorer school success has been related to an increased risk of unintentional injuries [27] and in longitudinal studies, poor academic achievement in primary and secondary education followed by low educational attainment in later life have increased the risk of injury deaths [7, 12]. Health compromising behavior has been linked to poorer school success, too [28].

Follow-up studies from adolescence to adulthood are few and understanding of the impact of health behaviors, health and socioeconomic circumstances in adolescence on later injury deaths is scarce. Here, we aim to investigate the influence of health behavior, health, sociodemographic factors of the family, and school success in adolescence on injury death in a large cohort of Finnish adolescents with an average 26-year follow-up. Our main interest was to investigate whether risky health behavior, poor perceived health, poor school success, and low family SES in adolescence associate with later injury deaths.

## Methods

### Baseline data

In this longitudinal study, we used both questionnaire and register data to examine possible risk factors for injury deaths. The baseline data were obtained from the Adolescent Health and Lifestyle Survey (AHLS) [29]. Since 1977, questionnaires have been sent biennially to all Finnish citizens aged 14, 16, or 18 years and born on certain days in June, July, or August. The samples were drawn from the Population Register Centre. Two re-inquiries were sent to non-responders. Data collected between the years 1981 and 1997 were used in the study. Data containing socioeconomic information on the respondent’s family (parental educational and occupational status) were obtained from the registries of Statistics Finland. Initial group in this study consisted of 60 278 individuals from which 47 326 responded to the survey. The overall response rate was 79%. In age-sex groups, the response rate among 14-year-old girls was 87% and 75% among boys, whereas among 16–18-year-old girls the rate was 86% and 69% among boys. Detailed information on response rates by survey year and age are reported at supplementary Table 1. One participant was excluded from the analyses due to negative follow-up time.

### Outcome variable

Follow-up data were obtained from the Finnish official Cause-of-Death Register (Statistics Finland), where causes of death have been classified by the International Classification of Diseases, 10th Revision (ICD-10) since 1996, and previously by the national classification of diseases 1987 and the International Classification of Diseases 8th Revision (ICD-8) [30]. The cause-of-death classification has 54 categories. Of these, the main categories for injury deaths are road traffic accidents, water traffic accidents, falls, drownings, poisonings, suicides, and homicides. Alcohol-related deaths were classified as deaths in which alcohol was not the primary cause of death but considered a contributing factor. The information related to deaths is based on death certificates. As data on alcohol as a contributing factor has only been available since 1987, information on five deaths that occurred prior to this date was not available.

The surveys were conducted in February and March and the individual follow-up started from the conclusion of each survey on April 30 each survey year. The endpoints were the occurrence of injury death, emigration, or termination of the study on December 31, 2018. The average follow-up time was 26 years, ranging from 0 to 37 years, and yielding a total of 1 209 933 person-years. The present study is an update of our previous study and, compared to that, our average follow-up time is 15 years longer [13].

### Explanatory variables

Three variables were used to measure health behavior in adolescence. First, smoking was described as daily use of tobacco with the categories yes, no. Second, drinking style was categorized as abstinence, occasional drinking (once a month but not drunkenness), recurrent drinking (more than once a month but rarely drunkenness), and recurring drunkenness (weekly). Third, the frequency of participation in sports clubs and leisure time physical exercise was categorized into three classes: never, 2 to 3 times a week or less, and 4 or more times a week.

Four variables from the AHLS describing respondents’ health were used. The respondents´ self-reported health was categorized as excellent, good, average, poor. Self-reported chronic diseases and disabilities were classified as yes or no. A summary variable was used for stress symptoms, which were stomachache, tension, irritability, sleep difficulty, headache, trembling of the hand, and feeling tired or weak. The categories were none, one, and two or more symptoms per day. BMI was used to evaluate obesity and cut-off values were set according to Cole’s criteria [31].

We had two variables describing family SES. The first variable indicated the highest attained educational level of the mother and father and comprised the following categories: both high, either one high, either one middle, both low. The second variable indicated the highest attained occupational status of the mother and father and comprised the following categories: both upper white-collar, either one upper white-collar, either one lower white-collar, either one blue-collar, both unknown.

Family structure was measured by family type with two categories: living with both parents, other. The urbanization level of the place of residence comprised five categories: capital area, large town, small town, village, sparsely populated rural municipality. Among 14-year-olds, school success was based on the respondents’ self-assessments of their success in relation to the class average. In 16–18-year-olds, the information was based on the success and educational path chosen after completion of comprehensive school education. Based on this information, a combined school success variable was formed with four categories: excellent (age 14: much better than average; 16–18: academic track and much better than average), good (age 14: better than average; 16–18: academic track and better than average / academic track and average/ vocational and much better or better than average), average (age 14: average; 16–18: academic track and slightly poorer than average / vocational and average or slightly poorer than average), and poor (age 14: slightly or much poorer than average; 16–18: academic track and much poorer than average / vocational and much poorer than average / not at school).

### Statistical methods

Cox regression model was used to analyze the associations between explanatory variables and injury death. Subgroups of 14-year-olds girls, 14-year-olds boys, 16–18-year-olds girls, and 16–18-year-olds boys were analyzed separately because the exposures and environments of these age groups vary, e.g., due to differences in type of school and adoption of risky health behaviors between the age groups. Sensitivity analyses were performed separately for suicides and other accidental deaths (suicides and homicides excluded) by gender. The results from the analyses were interpreted with adjusted hazard rations (aHR) and 95% confidence intervals (CI). Directed acyclic graphs (DAGs) were utilized to determine the variables for adjusting Cox’s models. DAGitty applies graph theory and causal inference algorithms to help automate the process, offering instant feedback on whether the chosen adjustments are sufficient. Helping researchers to ensure their causal conclusions are valid. DAGitty suggests the minimal adjustments needed to block all non-causal paths. [32] The selection of these variables was based on previously identified risk factors and hypothesized causal pathways. Lower family SES in childhood is associated with health compromising behaviors [15], lower participation in organized sports [16], poorer mental health [33], and adversities [34] during adolescence. Epitomizing SES, non-traditional family structure often reflect low material wealth too [35]. In addition, health compromising behaviors and family circumstances are associated with poorer school success [28, 36]. The models were created using the free online tool DAGitty (dagitty.net)**.** In the figures, pink arrows represent biasing paths, and green arrows represent causal paths. Green ellipse with triangle inside represents exposure and blue ellipses with line inside represent outcome. Blank ellipses represent following: ancestor of exposure (green), ancestor of outcome (blue), and ancestor of exposure and outcome (pink). (Supplementary Figs. 1–13). [37] Statistical analyses were performed with R version 4.0.5 (R Foundation for Statistical Computing, Vienna, Austria) using the Survival package.

## Results

During the mean 26-year follow-up period, 550 injury deaths occurred. The mean age at the time of death was 30 years (min 15, max 54) (Table 1). Men were more likely to die, as the number of deaths were 432 (79%) in men, whereas the corresponding figure for women was 118 (21%). The leading cause-of-death was suicide in all subgroups. Of all deaths, 170 (31%) were alcohol related (Supplementary Table 2).

**Table 1:** Basic information by age-sex subgroups

Subgroup	Mean follow-up time (years)	Mean age at the end of follow-up (years)	Mean age of death (years)	Sample size (n)	Injury deaths (n)
	(min, max)	(min, max)	(min, max)		
14-year-old girls	25 (0, 31)	41 (15, 46)	26 (15, 40)	8 303	35
14-year-old boys	25 (0, 31)	40 (15, 46)	29 (15, 46)	7 467	151
16–18-year-old girls	26 (0, 37)	44 (17, 56)	31 (17, 45)	17 248	83
16–18-year-old boys	26 (0, 37)	44 (17, 56)	31 (17, 54)	14 307	281
All	26 (0, 37)	43 (15, 56)	30 (15, 54)	47 325	550

In the Cox’s analysis, among 14-year-old girls and boys drinking style (in girls recurring drunkenness aHR 4.35 CI 1.00–19.02 and in boys recurrent drinking aHR 2.09, CI 1.17–3.74; recurring drunkenness aHR 4.02, CI 1.62–10.00) and not living with both parents (in girls aHR 2.06, CI 1.02–4.15 and in boys aHR 1.69, CI 1.19–2.41) increased the odds of injury death. Also, among 14-year-old boys poor school success (average aHR 3.06, CI 1.32–7.08; poor aHR 3.87, CI 1.33–11.26), low parental educational level (both parents’ low aHR 3.44, CI 1.06–11.19) and occupational status (both unknown aHR 2.14, CI 1.08–4.25), and poorer perceived health (good 1.49, CI 1.03–2.17) was associated with injury death. (Table 2).

**Table 2:** Adjusted hazard ratios (aHR) and confidence intervals (CI) for injury deaths by age-sex subgroups

	14-year-old girls			14-year-old boys		16–18-year-old girls		16–18-year-old boys	
	aHR	CI		aHR	CI	aHR	CI	aHR	CI
Daily use of tobacco ^a^									
No	1			1		1		1	
Yes	2.27	0.88–5.86		1.04	0.63–1.69	2.00	1.21–3.33	1.86	1.42–2.44
Drinking style ^b^									
Abstinence	1			1		1		1	
Occasional drinking	1.24	0.52–2.64		1.43	0.94–2.16	0.66	0.38–1.14	0.92	0.66–1.29
Recurrent drinking	1.00	0.28–3.58		2.09	1.17–3.74	1.03	0.53–1.99	1.28	0.89–1.83
Recurring drunkenness	4.35	1.00–19.02		4.02	1.62–10.00	2.63	1.13–6.13	1.70	1.07–2.71
Physical activity leisure time ^c^									
Never	1			1		1		1	
2 to 3 times a week	0.82	0.19–3.49		0.88	0.43–1.83	0.67	0.29–1.55	0.78	0.50–1.22
4 or more times a week	0.66	0.14–3.21		1.03	0.48–2.20	0.32	0.11–0.92	0.83	0.51–1.36
Physical activity in sports clubs ^d^									
Never	1			1		1		1	
2 to 3 times a week	0.70	0.35–1.45		1.03	0.72–1.46	0.25	0.11–0.58	0.99	0.75–1.30
4 or more times a week	0.00			0.84	0.51–1.40	0.46	0.11–1.90	0.77	0.50–1.21
Overweight e									
No	1			1		1		1	
Yes	0.94	0.28–3.10		0.85	0.50–1.43	1.80	0.92–3.50	0.73	0.49–1.10
Chronic disease or disability ^e^									
No	1			1		1		1	
Yes	1.36	0.48–3.87		0.86	0.45–1.64	0.91	0.42–1.99	1.39	0.93–2.06
Perceived health ^f^									
Excellent	1			1		1		1	
Good	1.08	0.47–2.49		1.49	1.03–2.17	1.01	0.54–1.89	1.00	0.75–1.35
Average or worse	1.14	0.40–3.26		1.37	0.79–2.39	1.39	0.71–2.74	1.50	1.06–2.13
Number of daily stress symptoms ^g^									
0	1			1		1		1	
1	1.34	0.58–3.11		1.43	0.90–2.27	2.26	1.36–3.78	1.44	1.02–2.03
2 +	1.35	0.47–3.91		1.13	0.55–2.32	2.34	1.32–4.14	2.07	1.39–3.07
School success ^h^									
Excellent	1			1		1		1	
Good	0.71	0.19–2.66		2.29	0.94–5.54	0.68	0.34–1.36	1.20	0.64–2.21
Average	1.68	0.54–5.16		3.06	1.32–7.08	0.65	0.28–1.50	1.91	1.04–3.53
Poor	2.97	0.47–18.76		3.87	1.33–11.26	0.79	0.32–1.93	1.80	0.90–3.22
Parental educational level									
Both parents’ high	1			1		1		1	
Either one high	1.93	0.22–17.29		1.67	0.47–5.97	0.93	0.37–2.29	0.71	0.34–1.47
Either one middle	1.91	0.26–14.19		3.08	0.98–9.70	0.49	0.22–1.09	1.06	0.59–1.90
Both parents’ low	2.52	0.32–20.14		3.44	1.06–11.19	0.52	0.22–1.23	1.75	0.96–3.18
Parental occupational status ^i^									
Both upper white collar	1			1		1		1	
Either one upper white collar	0.70	0.24–2.09		1.70	0.86–3.36	1.06	0.52–2.15	1.07	0.71–1.61
Either one lower white collar	0.74	0.26–2.16		1.78	0.91–3.48	0.72	0.32–1.62	1.08	0.72–1.59
Either one blue collar	1.07	0.25–4.51		1.69	0.69–4.13	1.97	0.72–5.43	1.55	0.90–2.67
Both unknown	0.69	0.21–1.90		2.14	1.08–4.25	1.61	0.76–3.42	1.13	0.75–1.70
Family structure									
Living with both parents	1			1		1		1	
Other	2.06	1.02–4.15		1.69	1.19–2.41	1.49	0.94–2.35	1.49	1.16–1.93
Urbanization level of residence									
Capital area	1			1		1		1	
Large town	0.35	0.10–1.25		0.94	0.48–1.83	0.52	0.25–1.07	1.27	0.73–2.22
Small town	0.42	0.16–1.14		0.74	0.40–1.38	0.35	0.18–0.68	1.10	0.66–1.85
Village	0.57	0.21–1.53		0.89	0.48–1.67	0.36	0.18–0.74	1.28	0.75–2.17
Sparsely populated rural municipality	0.18	0.04–0.90		0.85	0.43–1.64	0.54	0.26–1.14	1.37	0.80–2.37

^a^Adjusted by drinking style and family SES

^b^Adjusted by smoking and family SES

^c^Adjusted by physical activity in sports clubs and family SES

^d^Adjusted by leisure time physical activity and family SES

^e^Adjusted by leisure time physical activity and physical activity in sports clubs

^f^Adjusted by leisure time physical activity, physical activity in sports clubs and stress symptoms

^g^Adjusted by family SES

^h^Adjusted by family SES and risky behavior

^i^Adjusted by parental educational level

Among 16–18-year-old girls and boys daily use of tobacco (in girls aHR 2.00, CI 1.21–3.33 and in boys aHR 1.86, CI 1.42–2.44), drinking style (in girls recurring drunkenness aHR 2.63, CI 1.13–6.13 and in boys aHR 1.70, CI 1.07–2.71), and the number of stress symptoms (in girls one symptom/day aHR 2.26, CI 1.36–3.78; two or more symptoms/day aHR 2.34, CI 1.32–4.14 and in boys one symptom/day aHR 1.44, CI 1.02–2.03; two or more symptoms/day aHR 2.07, CI1.39–3.07) increased the risk of injury death. Also, among 16–18-year-old boys poorer perceived health (average or worse aHR 1.50, CI 1.06–2.13), poorer school success (average aHR 1.91, CI 1.04–3.53), and not living with both parents (aHR 1.49, CI 1.16–1.93) was associated with injury death. (Table 2) In the additional sensitivity analyses it was found that stress symptoms were associated more strongly with suicides (in girls two or more symptoms/day aHR 2.88, CI 1.51–5.52 and in boys one symptom/day aHR 1.52, CI 1.03–2.24; two or more symptoms/day aHR 1.80, CI 1.10–2.93) compared to other injury deaths (in girls one symptom/day aHR 2.22, CI 1.22–4.04) (Supplementary Table 3 and 4).

## Discussion

In this study, the main findings were the associations between injury death and health compromising behaviors (smoking and alcohol use), stress symptoms, and non-traditional family structure. Also, in boys’ an association between poor school success and injury death was observed and was stronger in younger age group.

An evident association was observed in both age groups between alcohol use and injury death. Recurring drunkenness increased the risk of injury death by up to fourfold, indicating particularly strong effect. Additionally, in the 16–18-year-olds, smoking was strongly associated with injury death (aHR ~ 2.00) highlighting its substantial impact as well. Both of these epitomizes risky health behaviors that has been previously linked with increased risk of injury death [13, 20, 38]. The use of alcohol can directly increase the risk for accidents [9] and alcohol use in adolescence may reflect on an individual’s later drinking behavior. Also influencing health behaviors, active exercise in adolescence was not an evident protective factor. However, sports have been associated with greater psychosocial health, particularly in team sports. Therefore, health enhancing behavior may steer adolescents away from risk-taking behavior and have positive long term effects on later health [39, 40].

The number of daily stress symptoms in adolescence were linked to later injury death with strong effect as the risk over doubled in older age groups. Our findings were in line with those of previous studies, where experiencing stress was associated with an increased risk for injury death [8, 13]. Currently, mental exhaustion and experiencing stress is a growing problem that may reflect suicidality in adolescence and later in life [7, 41]. This observation is also supported by our findings as the association was stronger in the sensitivity analyses for suicides compared to other injury deaths. Unfortunately, due to low number of deaths in our age-sex subgroups we were not able to conduct sensitivity analyses by age-sex subgroups. Additionally, perceived health was associated with injury death among boys, though the association was not entirely consistent. However, the impact on the risk of injury death was moderate (aHR ~ 1.5). As perceived health is an individualistic sum of several factors valuable to the index person, comprising both protective and predisposing factors, this may explain our finding between perceived health and injury death. In contrast to the findings of our study, there is also contradictory evidence of an association between poor perceived health and suicidality [42].

Adolescents from low SES backgrounds and non-traditional family structures had a higher risk of injury death, particularly boys. The effect of the risk was strong in low parental education and occupation in younger boys and moderate but consistent in non-traditional family structure. Our findings of a positive association between variables measuring family SES and injury death are in line with a Swedish study, where the impact of low family SES was stronger among boys [24]. Also, adolescent boys might be more sensitive to the influences of the family environment, or these factors may, in turn, increase their risk-taking behavior. The observed association between non-traditional family structure and injury death may reflect adversities, such as parental divorce, causing stress in adolescents’ life [43] and further leading to the adoption of negative health behaviors [44] or suicidality [45]. Suicides were the largest injury category in this study and the supplementary analyses showed stronger association between lower family SES and injury death in boys when only suicides were analyzed. Indeed, a Danish study has reported an association between low childhood family income and suicidality in later life [46]. Although we observed a strong (aHR up to 3.87) association between poor school success and injury death in boys, the association was not as evident in girls. Our finding is, however, in line with previous studies in which low educational attainment and occupational status later in life have been linked with suicides and injury deaths [7, 47]. Unfortunately, our study did not deal with the development of educational levels to the adulthood, which could have provided more data on the effects of education.

The main strength of this study was the large nationwide sample that comprehensively represented Finnish adolescents. The large representative sample and high response rates enhance the generalizability of the results to the broader population. The findings have significant public health implications, emphasizing the need for early intervention and preventive strategies to reduce injury deaths. The strongest associations were found for recurring drunkenness (aHR up to 4.35) suggesting a particularly high-risk behavior that requires immediate attention. While effect sizes around 1.5–2.0 represent moderate risks, they remain highly significant from a public health perspective due to their broad population impact. However, potential cultural or structural differences should still be considered in other sociocultural and healthcare contexts. Also, the long follow-up period increased information on the effects of conditions in adolescence on the health of the adolescents when they reach adulthood. This study also has its limitations. Response rates were slightly lower in boys compared to girls. The long follow-up period may also cause bias, as new exposures can either strengthen or weaken the association between the measured exposure and outcome. Also, as our birth cohorts were distant from each other (1981 to 1997) there might have been differences in the environmental factors influencing the adoption of health behaviors (e.g. smoking). Also, the possible problem of multiple testing and type 1 error was discussed. However, after careful consideration, authors chose not to apply formal multiple testing corrections, since it may be too conservative, and in this case would potentially lead to potential type 2 error. Both the possible type 1 error and the type 2 error may have an impact to the robustness and accuracy of the conclusions made.

## Conclusion

This study confirmed results from previous research regarding the association between health behaviors and health on later risk for injury death. More support should be allocated to adolescents who drift into negative health behaviors and struggle with stress.

## Supplementary Information

Supplementary Material 1. Supplementary Table 1. Number of participants and response rates by survey year and age.

Supplementary Material 2. Supplementary Table 2. Number of deaths in subgroups by alcohol use.

Supplementary Material 3. Supplementary Table 3. Sensitivity analysis for injury deaths in girls and boys. Suicides and homicides excluded.

Supplementary Material 4. Supplementary Table 4. Sensitivity analysis for suicides in girls and boys.

Supplementary Material 5. Supplementary Fig. 1. DAG: Smoking and the risk of injury death.

Supplementary Material 6. Supplementary Fig. 2. DAG: Alcohol use and the risk of injury death.

Supplementary Material 7. Supplementary Fig. 3. DAG: Leisure time physical activity and the risk of injury death.

Supplementary Material 8. Supplementary Fig. 4. DAG: Physical activity in sports clubs and the risk of injury death.

Supplementary Material 9. Supplementary Fig. 5. DAG: Overweight and the risk of injury death.

Supplementary Material 10. Supplementary Fig. 6. DAG: Chronic disease and the risk of injury death.

Supplementary Material 11. Supplementary Fig. 7. DAG: Perceived health and the risk of injury death.

Supplementary Material 12. Supplementary Fig. 8. DAG: Stress symptoms and the risk of injury death.

Supplementary Material 13. Supplementary Fig. 9. DAG: School success and the risk of injury death.

Supplementary Material 14. Supplementary Fig. 10. DAG: Parental educational level and the risk of injury death.

Supplementary Material 15. Supplementary Fig. 11. DAG: Parental occupational status and the risk of injury death.

Supplementary Material 16. Supplementary Fig. 12. DAG: Family structure and the risk of injury death.

Supplementary Material 17. Supplementary Fig. 13 DAG: Urbanization level of residence and the risk of injury death.

## Data Availability

Due to existing Finnish data legislation, sharing the data is not possible.

## Abbreviations

AHLS: Adolescent Health and Lifestyle Survey
SES: Socioeconomic status
DAG: Directed acyclic graph
aHR: Adjusted hazard ratio
CI: Confidence interval
