# Supplementary material for: Health behaviors, health, sociodemographic factors, and school success in adolescence as risk factors for injury deaths: a longitudinal study
Source: BMC Public Health. 2025 May 29;25:1981. doi: 10.1186/s12889-025-23214-0 (PMC12121201; doi:10.1186/s12889-025-23214-0)
Supplement: Supplementary file 1 — Supplementary Material 1. Supplementary Table 1. Number of participants and response rates by survey year and age. [file 12889_2025_23214_MOESM1_ESM.docx]

**Supplementary table 1.** Number of participants and response rates by survey year and age.

| Survey year | 14-year-old girls, n | 14-year-old boys, n | 16-year-old girls, n | 16-year-old boys, n | 18-year-old girls, n | 18-year-old boys, n |
| --- | --- | --- | --- | --- | --- | --- |
|  | (response rate) | response rate) | response rate) | (response rate) | (response rate) | (response rate) |
| 1981 | 0 | 0 | 3 (67%) | 5 (60%) | 578 (89 %) | 615 (81%) |
| 1985 | 0 | 0 | 3 (100%) | 3 (33%) | 552 (83%) | 583 (68%) |
| 1987 | 1562 (90%) | 1604 (83%) | 1633 (89%) | 1698 (80%) | 1412 (89%) | 1344 (74%) |
| 1989 | 476 (90%) | 468 (75%) | 456 (78%) | 506 (70%) | 494 (81%) | 512 (63%) |
| 1991 | 2107 (87%) | 2165 (74%) | 2186 (87%) | 2179 (71%) | 1967 (82%) | 2073 (61%) |
| 1993 | 2273 (88%) | 2465 (75%) | 2218 (87%) | 2319 (71%) | 2121 (84%) | 2162 (67%) |
| 1995 | 1528 (85%) | 1556 (75%) | 1664 (88%) | 1691 (72%) | 1520 (86%) | 1594 (67%) |
| 1997 | 1586 (84%) | 1677 (69 %) | 1583 (87%) | 1647 (68%) | 1691 (84%) | 1799 (60%) |
