# Supplementary material for: Health behaviors, health, sociodemographic factors, and school success in adolescence as risk factors for injury deaths: a longitudinal study
Source: BMC Public Health. 2025 May 29;25:1981. doi: 10.1186/s12889-025-23214-0 (PMC12121201; doi:10.1186/s12889-025-23214-0)
Supplement: Supplementary file 2 — Supplementary Material 2. Supplementary Table 2. Number of deaths in subgroups by alcohol use. [file 12889_2025_23214_MOESM2_ESM.docx]

| **Supplementary table 2.** Number of deaths in subgroups by alcohol use | | | | |  |  |
| --- | --- | --- | --- | --- | --- | --- |
| Injury category | | Subgroup girls aged 14 (alcohol-related) | Subgroup boys aged 14 (alcohol-related) | Subgroup girls aged 16-18 (alcohol-related) | Subgroup boys aged 16-18 (alcohol-related) | All (alcohol-related) |
| Unintentional injury | |  |  |  |  |  |
|  | Traffic accidents | 10 (1) | 30 (9) | 21 (2) | 49 (19) | 110 (31) |
|  | Water traffic accidents | 0 | 1 (1) | 0 | 3 (2) | 4 (3) |
|  | Falls | 0 | 5 (5) | 2 (2) | 8 (5) | 15 (12) |
|  | Drownings | 0 | 5 (3) | 1 (0) | 10 (4) | 16 (7) |
|  | Poisonings | 5 (0) | 19 (0) | 7 (0) | 35 (1) | 66 (1) |
|  | Others | 0 | 6 (3) | 6 (4) | 21 (6) | 33 (13) |
| Intentional injury | |  |  |  |  |  |
|  | Suicides | 17 (5) | 72 (30) | 38 (5) | 133 (46) | 260 (86) |
|  | Homicides | 1 (0) | 5 (1) | 5 (3) | 9 (5) | 20 (9) |
|  | Others | 2 (0) | 8 (5) | 2 (1) | 9 (2) | 21 (8) |
| Total |  | 35 (6) | 151 (57) | 82 (17) | 277 (90) | 545 (170) |
