# Supplementary material for: Health behaviors, health, sociodemographic factors, and school success in adolescence as risk factors for injury deaths: a longitudinal study
Source: BMC Public Health. 2025 May 29;25:1981. doi: 10.1186/s12889-025-23214-0 (PMC12121201; doi:10.1186/s12889-025-23214-0)
Supplement: Supplementary file 3 — Supplementary Material 3. Supplementary Table 3. Sensitivity analysis for injury deaths in girls and boys. Suicides and homicides excluded. [file 12889_2025_23214_MOESM3_ESM.docx]

**Supplementary table 3.** Sensitivity analysis for injury deaths in girls and boys. Suicides and homicides excluded.

|  |  | Girls |  | Boys |  |
| --- | --- | --- | --- | --- | --- |
|  |  | aHR | CI | aHR | CI |
| Daily use of tobacco ^a^ | | |  |  |  |
|  | No | 1 |  | 1 |  |
|  | Yes | 2.39 | 1.27-4.51 | 1.34 | 0.96-1.86 |
| Drinking style ^b^ | |  |  |  |  |
|  | Abstinence | 1 |  | 1 |  |
|  | Occasional drinking | 1.09 | 0.54-2.18 | 1.03 | 0.72-1.48 |
|  | Recurrent drinking | 1.75 | 0.77-3.95 | 1.63 | 1.09-2.44 |
|  | Recurring drunkenness | 2.63 | 0.79-8.78 | 2.38 | 1.37-4.13 |
| Physical activity leisure time ^c^ | | |  |  |  |
|  | Never | 1 |  | 1 |  |
|  | 2 to 3 times a week | 2.44 | 0.33-17.86 | 0.74 | 0.43-1.27 |
|  | 4 or more times a week | 2.31 | 0.30-18.03 | 0.94 | 0.53-1.69 |
| Physical activity in sports clubs ^d^ | |  |  |  |  |
|  | Never | 1 |  | 1 |  |
|  | 2 to 3 times a week | 0.54 | 0.27-1.09 | 1.11 | 0.82-1.51 |
|  | 4 or more times a week | 0.57 | 0.14-2.36 | 0.86 | 0.54-1.38 |
| Overweight ^e^ | |  |  |  |  |
|  | No | 1 |  | 1 |  |
|  | Yes | 1.90 | 0.85-4.23 | 0.81 | 0.52-1.28 |
| Chronic disease or disability ^e^ | | |  |  |  |
|  | No | 1 |  | 1 |  |
|  | Yes | 1.70 | 0.80-3.62 | 1.11 | 0.67-1.83 |
| Perceived health ^f^ | |  |  |  |  |
|  | Excellent | 1 |  | 1 |  |
|  | Good | 0.99 | 0.51-1.92 | 1.03 | 0.74-1.44 |
|  | Average or worse | 0.77 | 0.33-1.83 | 1.49 | 0.99-2.25 |
| Number of daily stress symptoms ^g^ | | | |  |  |
|  | 0 | 1 |  | 1 |  |
|  | 1 | 2.22 | 1.22-4.04 | 1.39 | 0.93-2.07 |
|  | 2+ | 1.26 | 0.52-3.01 | 1.60 | 0.96-2.68 |
| School success ^h^ | |  |  |  |  |
|  | Excellent | 1 |  | 1 |  |
|  | Good | 0.67 | 0.29-1.56 | 1.80 | 0.76-4.26 |
|  | Average | 0.70 | 0.28-1.80 | 4.34 | 1.89-9.99 |
|  | Poor | 0.88 | 0.28-2.74 | 3.36 | 1.37-8.24 |
| Parental educational level | | |  |  |  |
|  | Both parents’ high | 1 |  | 1 |  |
|  | Either one high | 0.94 | 0.32-2.75 | 0.93 | 0.40-2.18 |
|  | Either one middle | 0.48 | 0.18-1.23 | 1.33 | 0.65-2.73 |
|  | Both parents’ low | 0.52 | 0.18-1.47 | 1.87 | 0.90-3.89 |
| Parental occupational status ^i^ | | |  |  |  |
|  | Both upper white collar | 1 |  | 1 |  |
|  | Either one upper white collar | 0.46 | 0.18-1.14 | 1.23 | 0.76-2.00 |
|  | Either one lower white collar | 0.81 | 0.33-1.96 | 1.41 | 0.88-2.25 |
|  | Either one blue collar | 1.45 | 0.43-4.82 | 1.17 | 0.57-2.40 |
|  | Both unknown | 1.23 | 0.52-3.09 | 1.14 | 0.69-1.88 |
| Family structure | |  |  |  |  |
|  | Living with both parents | 1 |  | 1 |  |
|  | Other | 1.27 | 0.71-2.27 | 1.33 | 0.98-1.81 |
| Urbanization level of residence | | |  |  |  |
|  | Capital area | 1 |  | 1 |  |
|  | Large town | 0.17 | 0.06-0.46 | 1.03 | 0.55-1.95 |
|  | Small town | 0.27 | 0.14-0.53 | 1.04 | 0.59-1.85 |
|  | Village | 0.21 | 0.09-0.47 | 1.20 | 0.67-2.16 |
|  | Sparsely populated rural municipality | 0.22 | 0.08-0.56 | 1.63 | 0.63-2.14 |
| a Adjusted by drinking style and family SES | | | |  |  |
| b Adjusted by smoking and family SES | | | |  |  |
| c Adjusted by physical activity in sports clubs and family SES | | | | |  |
| d Adjusted by leisure time physical activity and family SES | | | | |  |
| e Adjusted by leisure time physical activity and physical activity in sports clubs | | | | | |
| f Adjusted by leisure time physical activity, physical activity in sports clubs and stress symptoms | | | | | |
| g Adjusted by family SES | | |  |  |  |
| h Adjusted by family SES and risky behavior | | | |  |  |
| i Adjusted by parental educational level | | | |  |  |
