# Supplementary material for: Health behaviors, health, sociodemographic factors, and school success in adolescence as risk factors for injury deaths: a longitudinal study
Source: BMC Public Health. 2025 May 29;25:1981. doi: 10.1186/s12889-025-23214-0 (PMC12121201; doi:10.1186/s12889-025-23214-0)
Supplement: Supplementary file 4 — Supplementary Material 4. Supplementary Table 4. Sensitivity analysis for suicides in girls and boys. [file 12889_2025_23214_MOESM4_ESM.docx]

**Supplementary table 4.** Sensitivity analysis for suicides in girls and boys.

|  |  | Girls |  | Boys |  |
| --- | --- | --- | --- | --- | --- |
|  |  | aHR | CI | aHR | CI |
| Daily use of tobacco ^a^ | | |  |  |  |
|  | No | 1 |  | 1 |  |
|  | Yes | 1.65 | 0.84-3.23 | 1.80 | 1.29-2.51 |
| Drinking style ^b^ | |  |  |  |  |
|  | Abstinence | 1 |  | 1 |  |
|  | Occasional drinking | 0.68 | 0.36-1.29 | 0.80 | 0.56-1.15 |
|  | Recurrent drinking | 0.77 | 0.32-1.87 | 1.07 | 0.71-1.61 |
|  | Recurring drunkenness | 2.60 | 0.87-7.76 | 1.39 | 0.78-2.47 |
| Physical activity leisure time ^c^ | |  |  |  |  |
|  | Never | 1 |  | 1 |  |
|  | 2 to 3 times a week | 0.42 | 0.19-0.94 | 0.95 | 0.54-1.66 |
|  | 4 or more times a week | 0.10 | 0.03-0.40 | 0.87 | 0.47-1.60 |
| Physical activity in sports clubs ^d^ | |  |  |  |  |
|  | Never | 1 |  | 1 |  |
|  | 2 to 3 times a week | 0.37 | 0.17-0.82 | 0.97 | 0.71-1.32 |
|  | 4 or more times a week | 0.00 |  | 0.67 | 0.40-1.13 |
| Overweight ^e^ | |  |  |  |  |
|  | No | 1 |  | 1 |  |
|  | Yes | 1.34 | 0.57-3.13 | 0.74 | 0.46-1.18 |
| Chronic disease or disability ^e^ | | |  |  |  |
|  | No | 1 |  | 1 |  |
|  | Yes | 0.57 | 0.18-1.84 | 1.39 | 0.88-2.19 |
| Perceived health ^f^ | |  |  |  |  |
|  | Excellent | 1 |  | 1 |  |
|  | Good | 1.12 | 0.50-2.51 | 1.28 | 0.91-1.79 |
|  | Average or worse | 1.92 | 0.82-4.46 | 1.43 | 0.93-2.20 |
| Number of daily stress symptoms ^g^ | | | |  |  |
|  | 0 | 1 |  | 1 |  |
|  | 1 | 1.76 | 0.90-3.42 | 1.52 | 1.03-2.24 |
|  | 2+ | 2.88 | 1.51-5.52 | 1.80 | 1.10-2.93 |
| School success ^h^ | |  |  |  |  |
|  | Excellent | 1 |  | 1 |  |
|  | Good | 0.76 | 0.31-1.85 | 1.25 | 0.67-2.35 |
|  | Average | 1.24 | 0.50-3.05 | 1.43 | 0.77-2.66 |
|  | Poor | 0.67 | 0.19-2.31 | 1.48 | 0.75-2.94 |
| Parental educational level | | |  |  |  |
|  | Both parents’ high | 1 |  | 1 |  |
|  | Either one high | 1.10 | 0.28-4.25 | 0.87 | 0.34-2.17 |
|  | Either one middle | 0.85 | 0.26-2.78 | 1.50 | 0.70-3.21 |
|  | Both parents’ low | 1.14 | 0.33-3.91 | 2.28 | 1.05-4.96 |
| Parental occupational status ^i^ | | |  |  |  |
|  | Both upper white collar | 1 |  | 1 |  |
|  | Either one upper white collar | 1.58 | 0.66-3.77 | 1.16 | 0.70-1.92 |
|  | Either one lower white collar | 0.66 | 0.24-1.80 | 1.07 | 0.65-1.76 |
|  | Either one blue collar | 2.00 | 0.62-6.49 | 1.97 | 1.05-3.70 |
|  | Both unknown | 1.23 | 0.48-3.18 | 1.59 | 0.97-2.61 |
| Family structure | |  |  |  |  |
|  | Living with both parents | 1 |  | 1 |  |
|  | Other | 2.05 | 1.20-3.51 | 1.66 | 1.23-2.23 |
| Urbanization level of residence | | |  |  |  |
|  | Capital area | 1 |  | 1 |  |
|  | Large town | 1.48 | 0.48-4.60 | 1.13 | 0.62-2.07 |
|  | Small town | 0.79 | 0.26-2.38 | 0.84 | 0.48-1.48 |
|  | Village | 1.17 | 0.39-3.52 | 1.09 | 0.61-1.92 |
|  | Sparsely populated rural municipality | 1.35 | 0.42-4.30 | 1.18 | 0.66-2.13 |
| a Adjusted by drinking style and family SES | | | |  |  |
| b Adjusted by smoking and family SES | | | |  |  |
| c Adjusted by physical activity in sports clubs and family SES | | | | |  |
| d Adjusted by leisure time physical activity and family SES | | | | |  |
| e Adjusted by leisure time physical activity and physical activity in sports clubs | | | | | |
| f Adjusted by leisure time physical activity, physical activity in sports clubs and stress symptoms | | | | | |
| g Adjusted by family SES | | |  |  |  |
| h Adjusted by family SES and risky behavior | | | |  |  |
| i Adjusted by parental educational level | | | |  |  |
