# Supplementary figures and images for: Health behaviors, health, sociodemographic factors, and school success in adolescence as risk factors for injury deaths: a longitudinal study
Source: BMC Public Health. 2025 May 29;25:1981. doi: 10.1186/s12889-025-23214-0 (PMC12121201; doi:10.1186/s12889-025-23214-0)

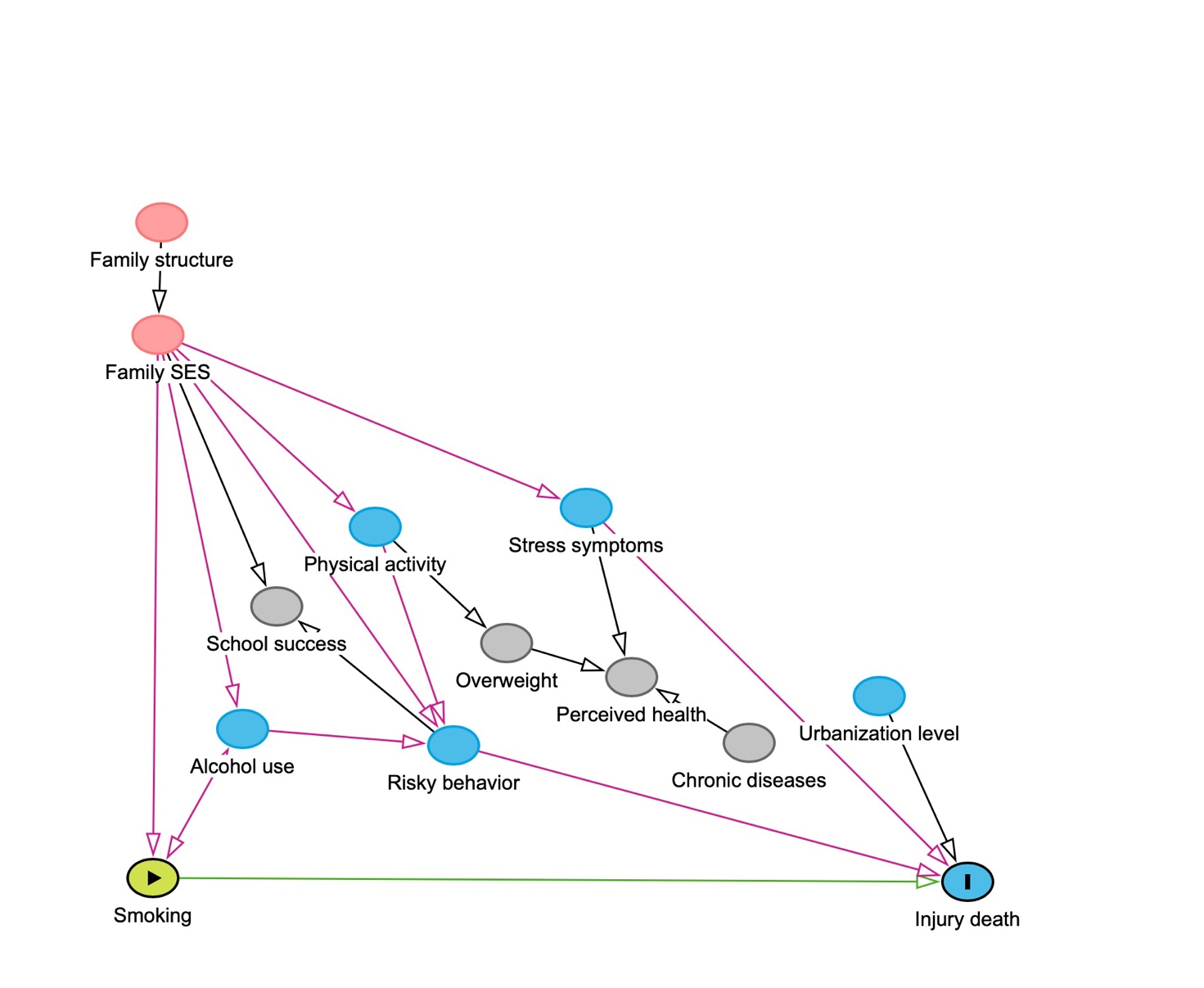


**Supplementary figure 1.** DAG:Smoking and the risk of injury death

Supplement: Supplementary file 5 — Supplementary Material 5. Supplementary Fig. 1. DAG: Smoking and the risk of injury death. [file 12889_2025_23214_MOESM5_ESM.docx]

**
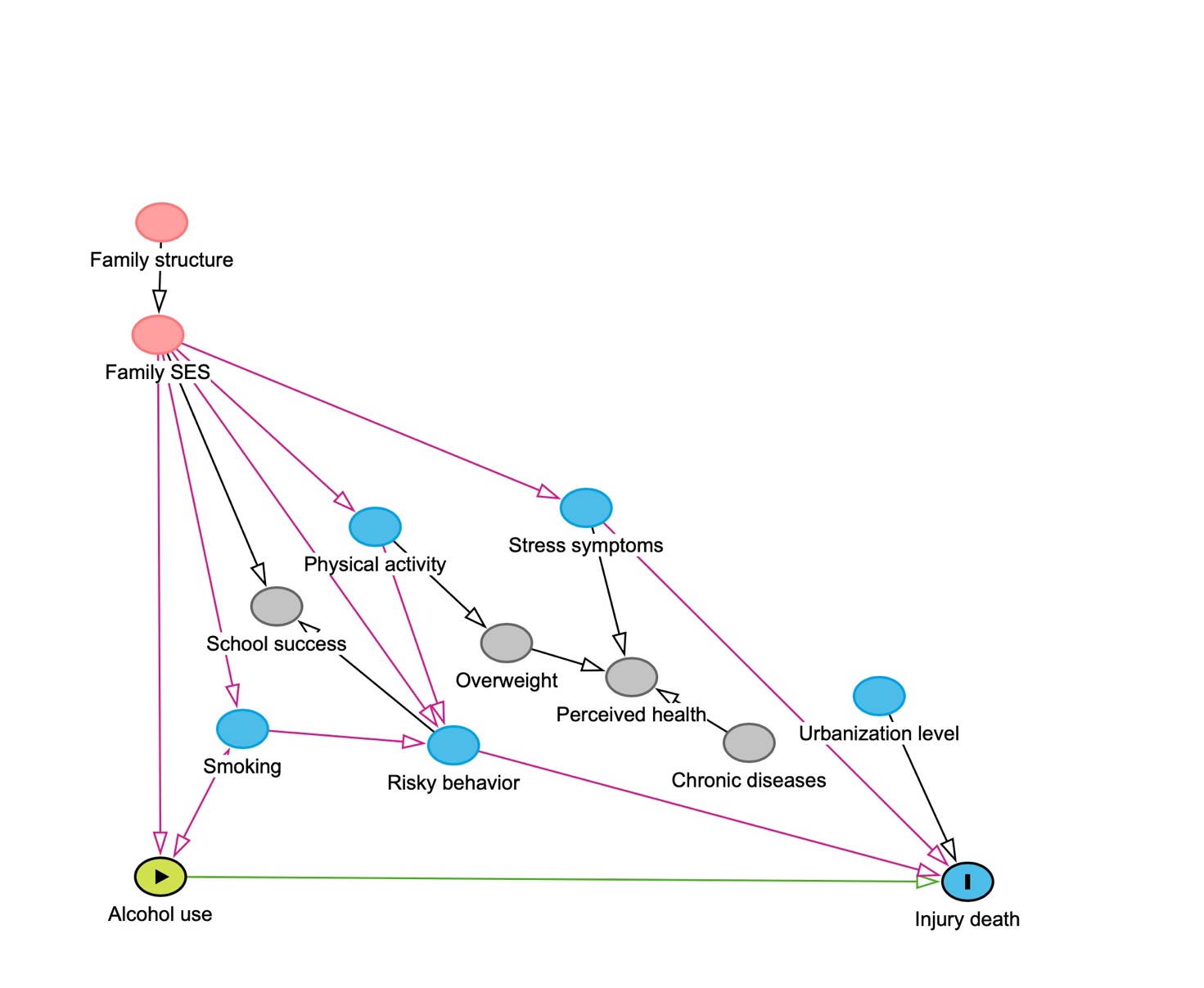
**

**Supplementary figure 2.** DAG: Alcohol use and the risk of injury death.

Supplement: Supplementary file 6 — Supplementary Material 6. Supplementary Fig. 2. DAG: Alcohol use and the risk of injury death. [file 12889_2025_23214_MOESM6_ESM.docx]

**
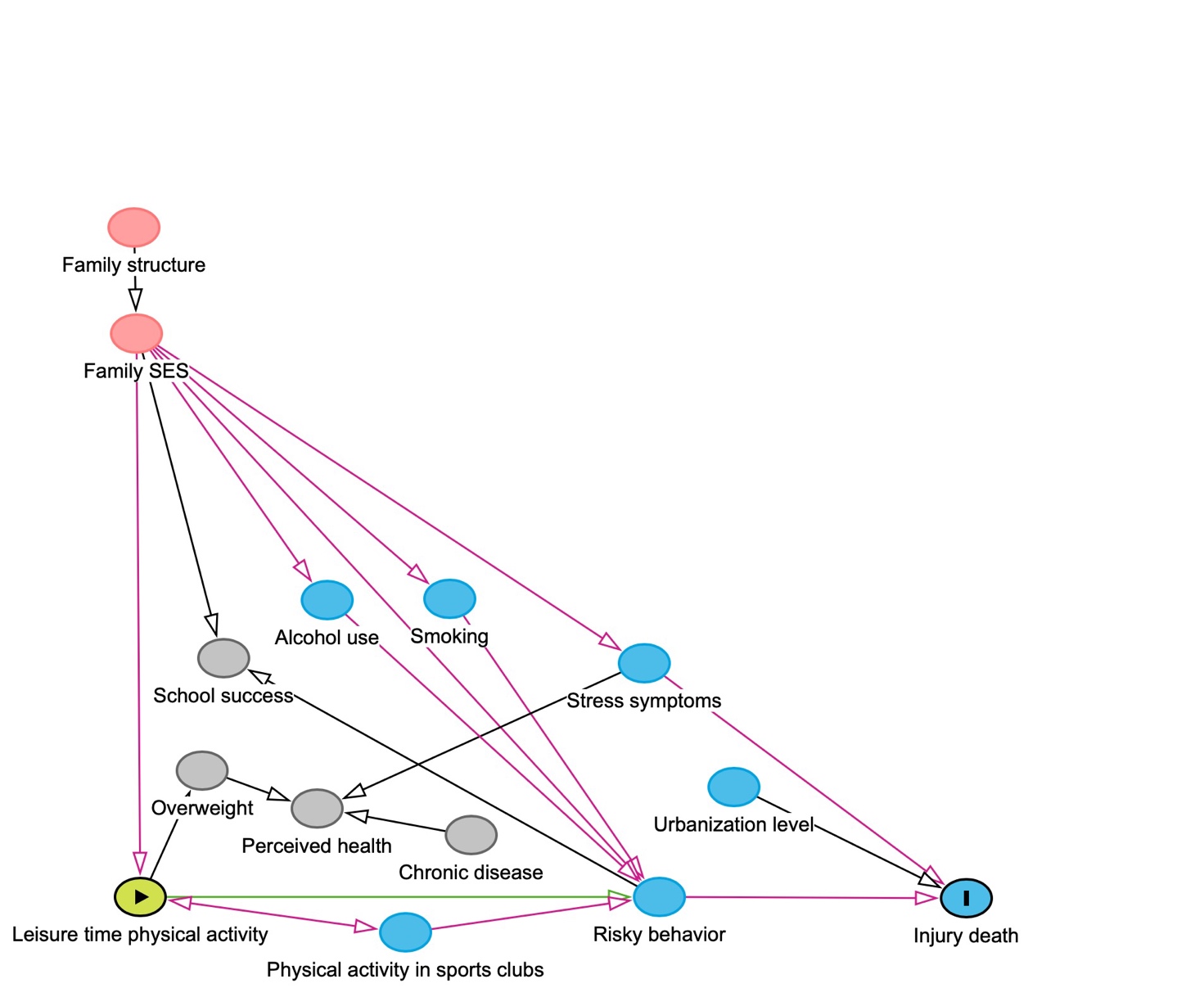
Supplementary figure 3.** DAG: Leisure time physical activity and the risk of injury death.

Supplement: Supplementary file 7 — Supplementary Material 7. Supplementary Fig. 3. DAG: Leisure time physical activity and the risk of injury death. [file 12889_2025_23214_MOESM7_ESM.docx]

**
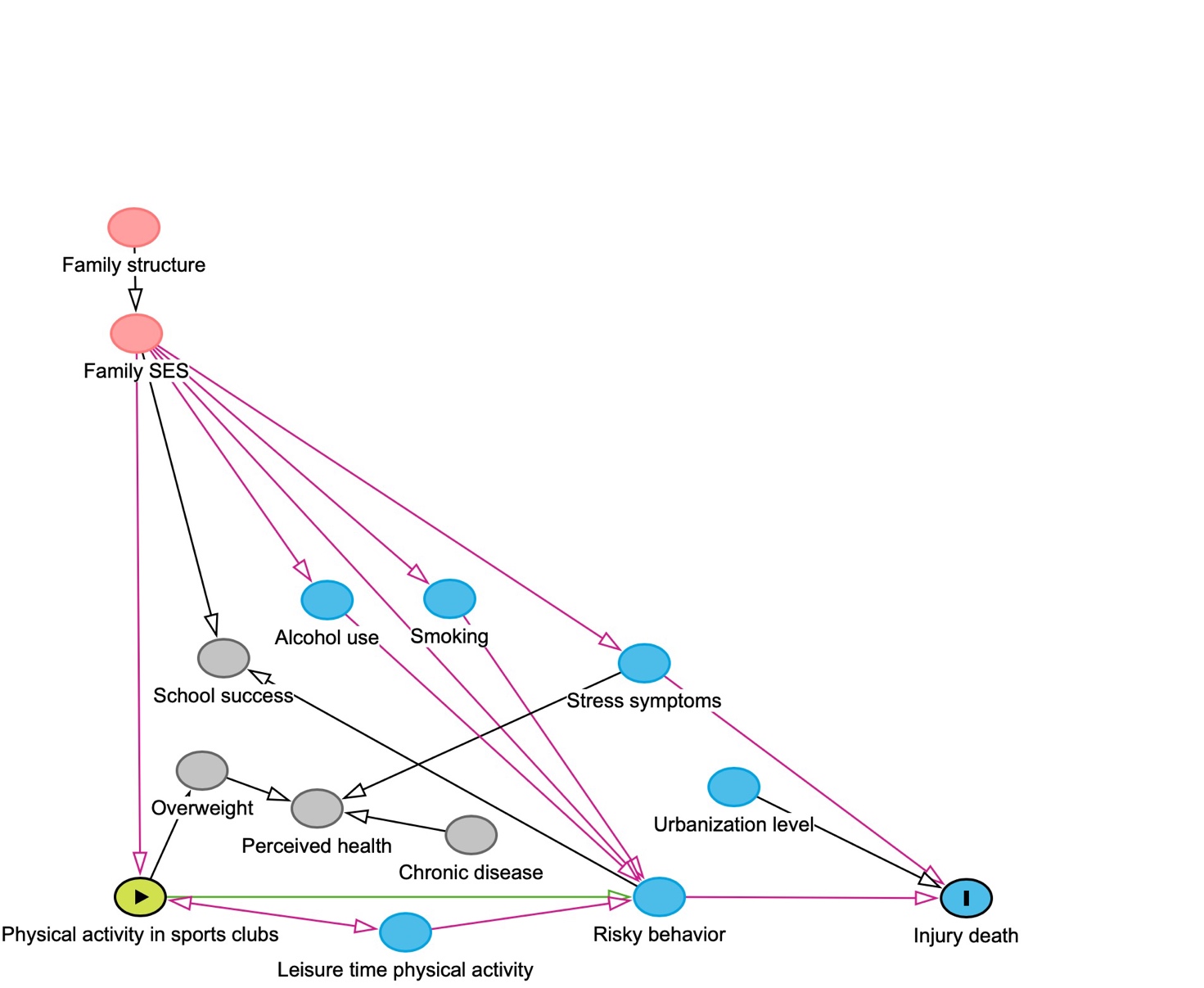
Supplementary figure 4.** DAG:Physical activity in sports clubs and the risk of injury death.

Supplement: Supplementary file 8 — Supplementary Material 8. Supplementary Fig. 4. DAG: Physical activity in sports clubs and the risk of injury death. [file 12889_2025_23214_MOESM8_ESM.docx]

**
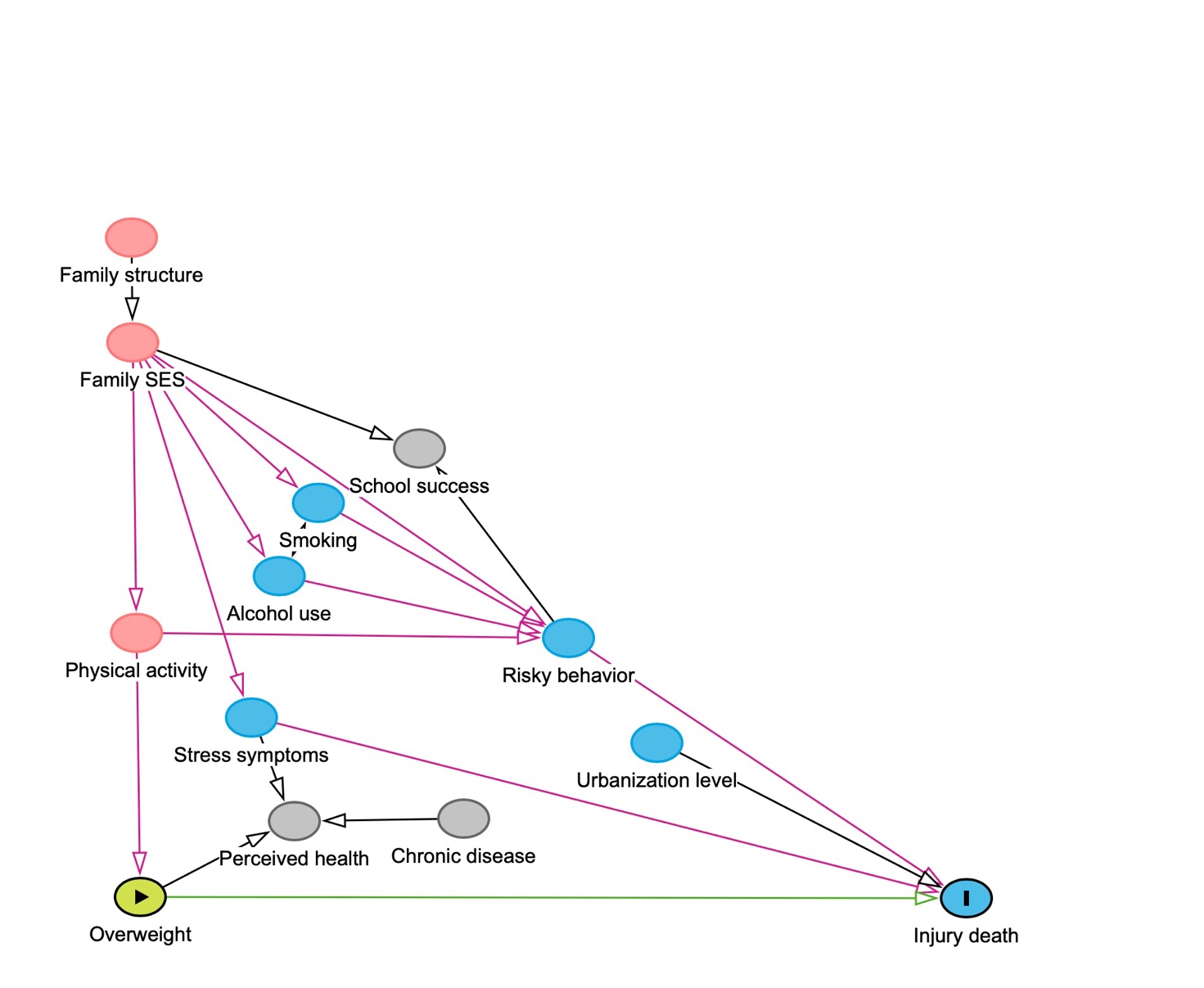
**

**Supplementary figure 5.** DAG:Overweight and the risk of injury death.

Supplement: Supplementary file 9 — Supplementary Material 9. Supplementary Fig. 5. DAG: Overweight and the risk of injury death. [file 12889_2025_23214_MOESM9_ESM.docx]

**
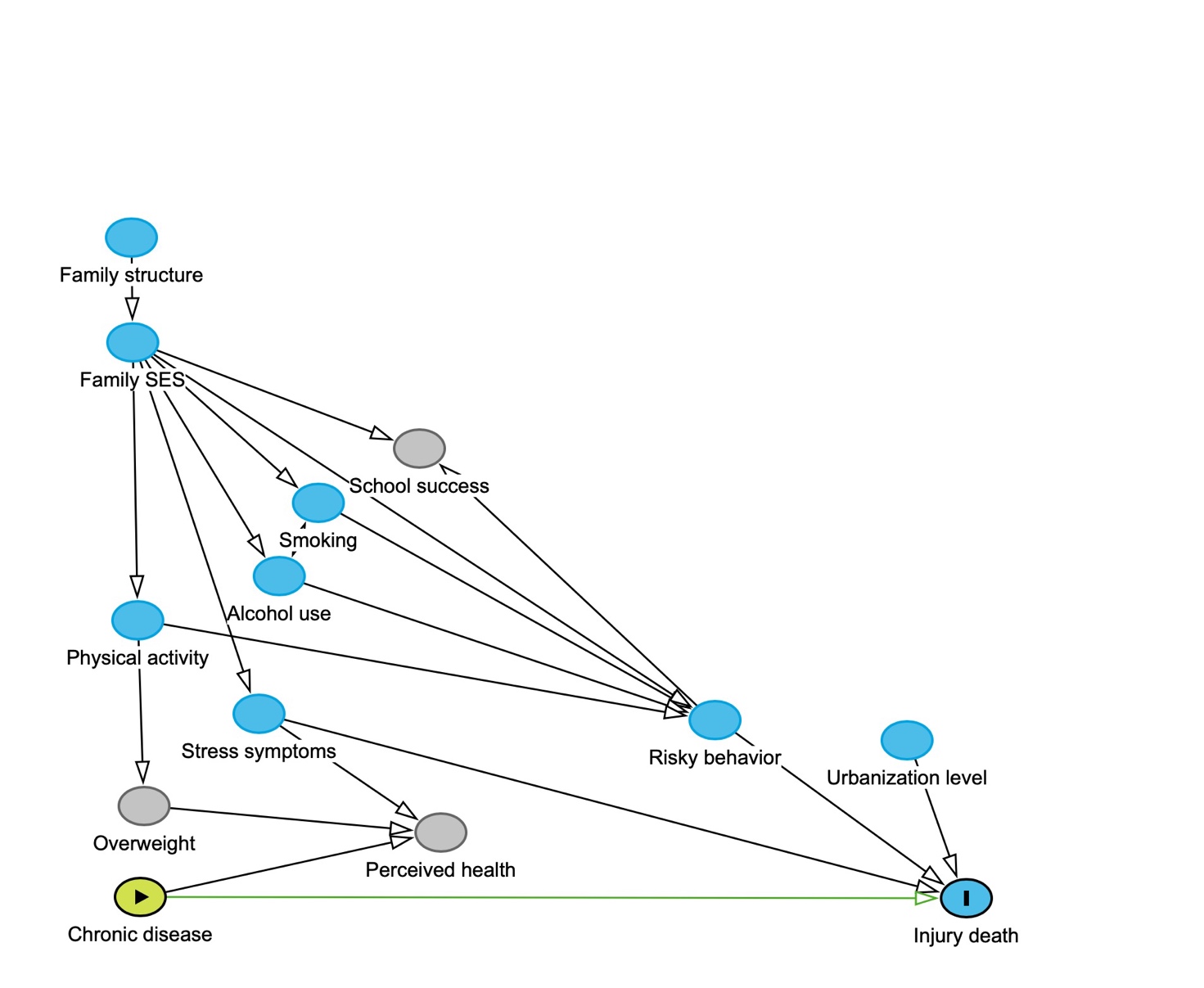
Supplementary figure 6.** DAG:Chronic disease and the risk of injury death.

Supplement: Supplementary file 10 — Supplementary Material 10. Supplementary Fig. 6. DAG: Chronic disease and the risk of injury death. [file 12889_2025_23214_MOESM10_ESM.docx]

**
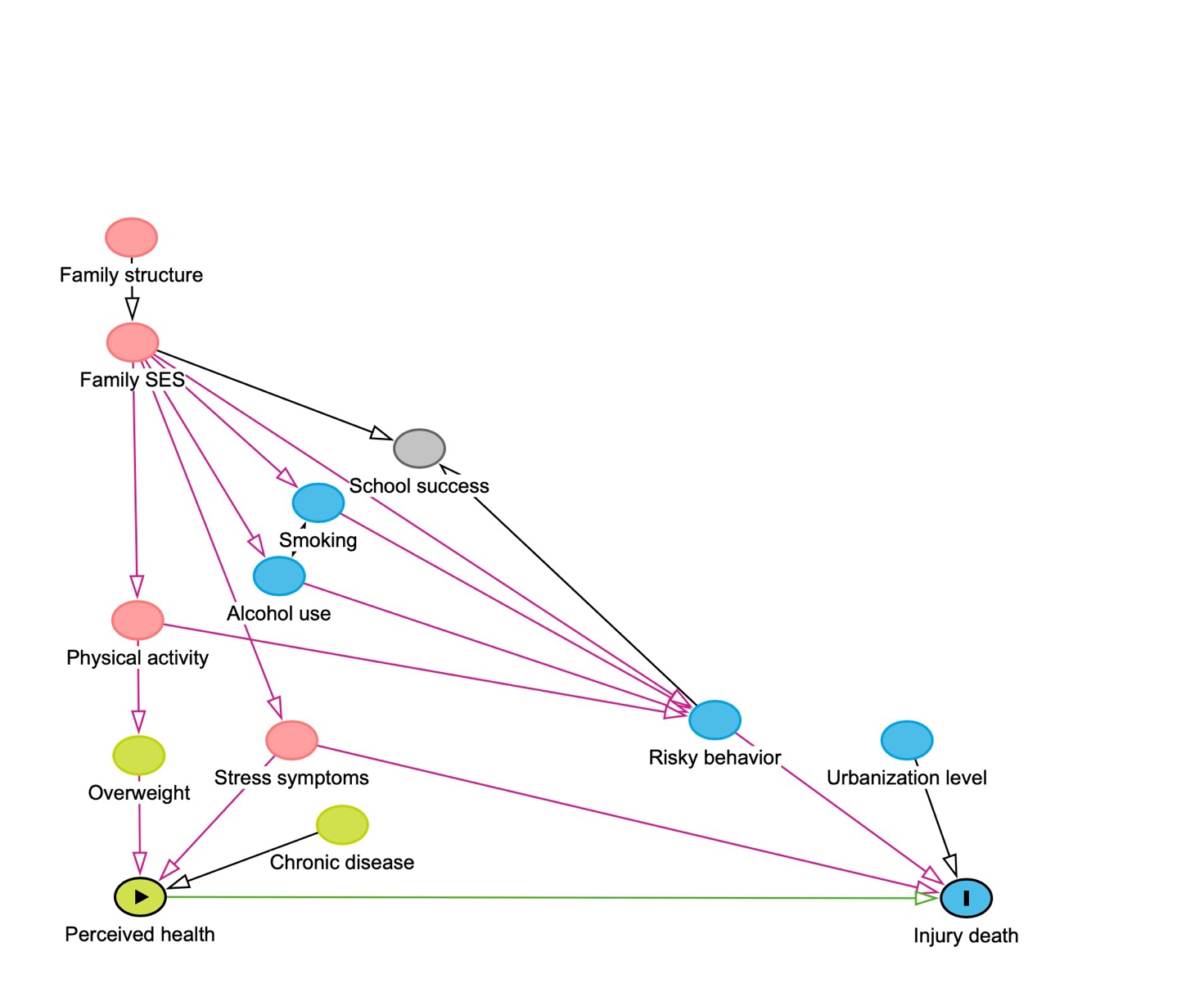
Supplementary figure 7.** DAG:Perceived health and the risk of injury death.

Supplement: Supplementary file 11 — Supplementary Material 11. Supplementary Fig. 7. DAG: Perceived health and the risk of injury death. [file 12889_2025_23214_MOESM11_ESM.docx]

**
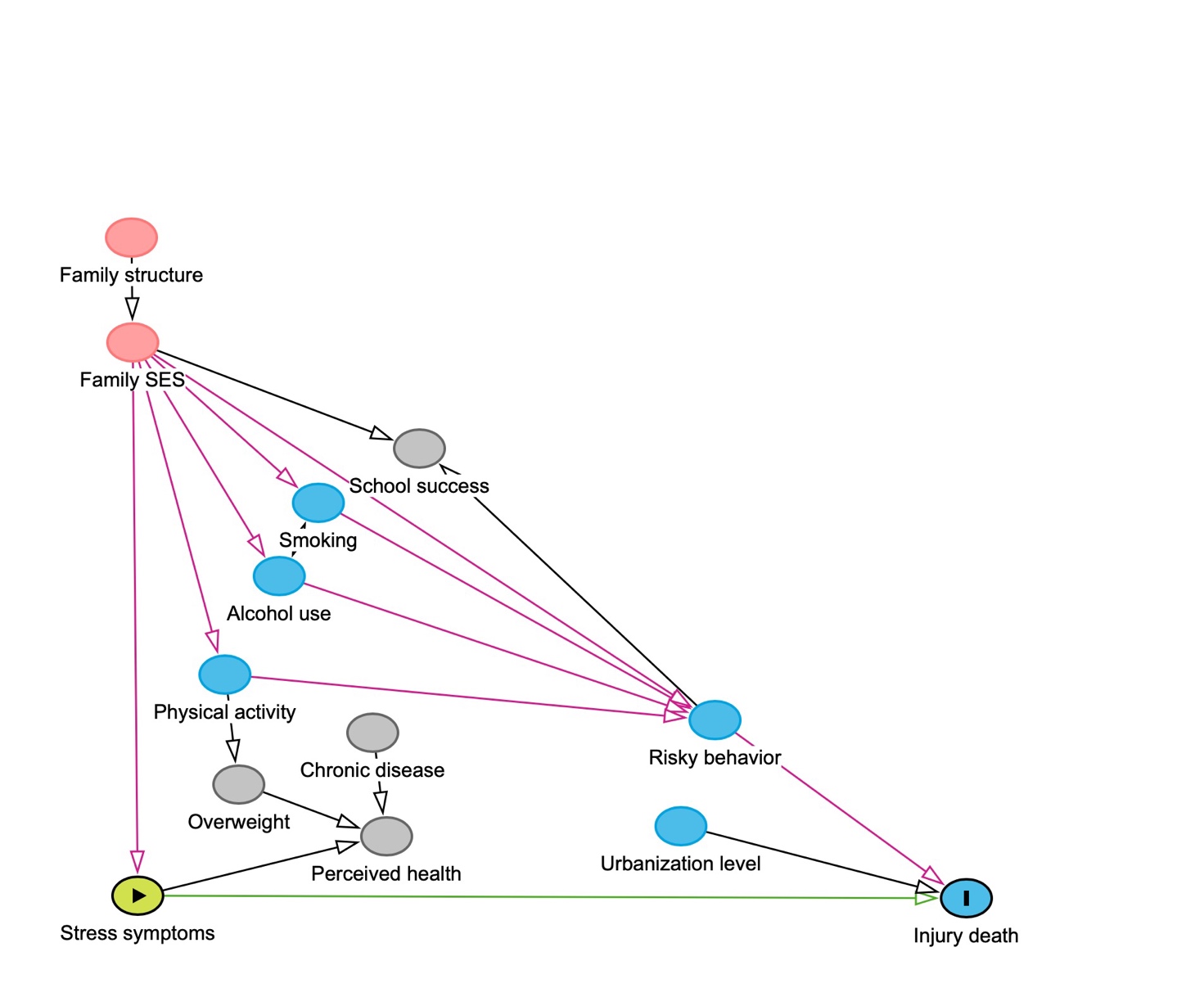
Supplementary figure 8.** DAG:Stress symptoms and the risk of injury death.

Supplement: Supplementary file 12 — Supplementary Material 12. Supplementary Fig. 8. DAG: Stress symptoms and the risk of injury death. [file 12889_2025_23214_MOESM12_ESM.docx]

**
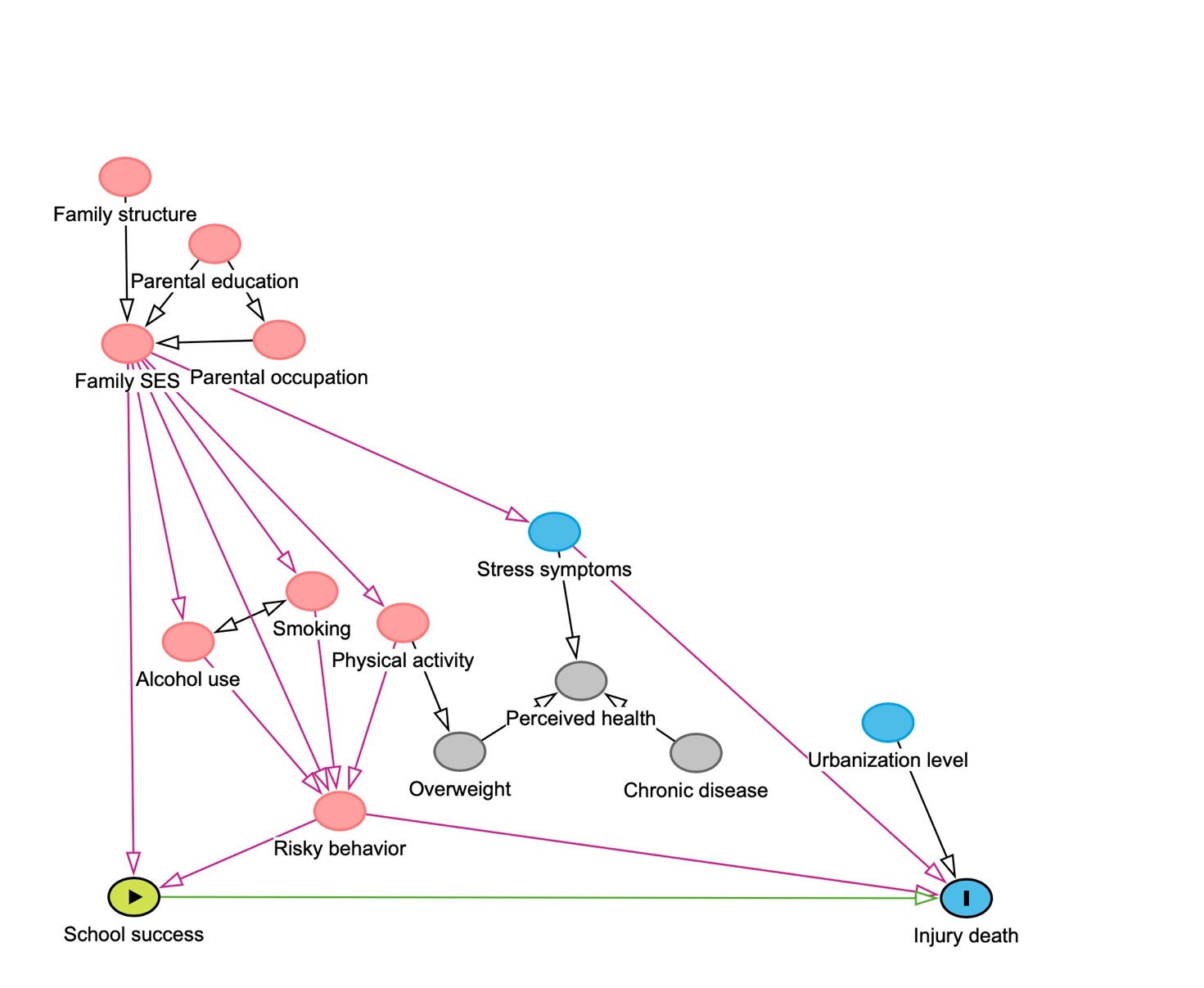
Supplementary figure 9.** DAG:School success and the risk of injury death.

Supplement: Supplementary file 13 — Supplementary Material 13. Supplementary Fig. 9. DAG: School success and the risk of injury death. [file 12889_2025_23214_MOESM13_ESM.docx]

**
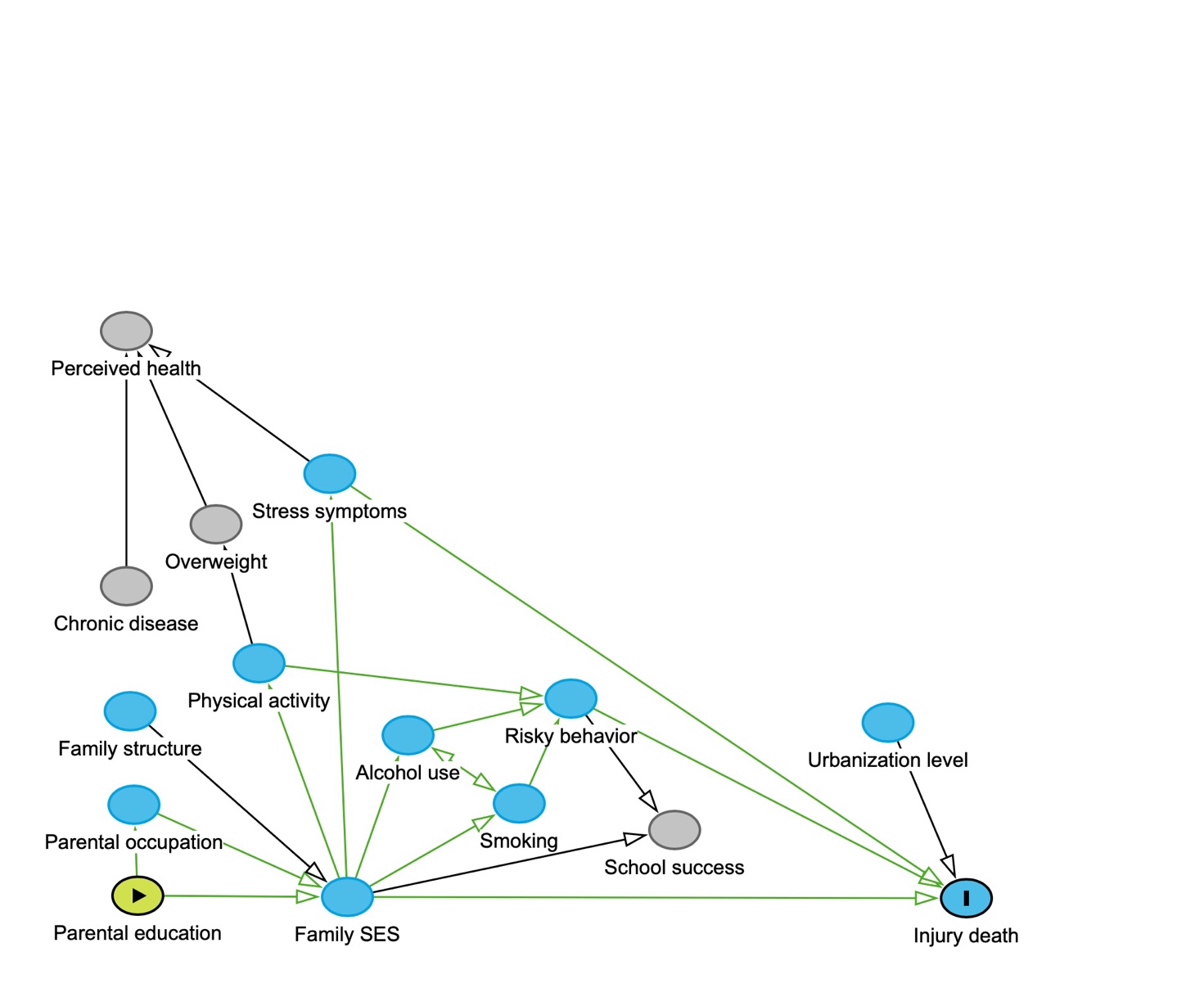
Supplementary figure 10.** DAG:Parental educational level and the risk of injury death.

Supplement: Supplementary file 14 — Supplementary Material 14. Supplementary Fig. 10. DAG: Parental educational level and the risk of injury death. [file 12889_2025_23214_MOESM14_ESM.docx]

**
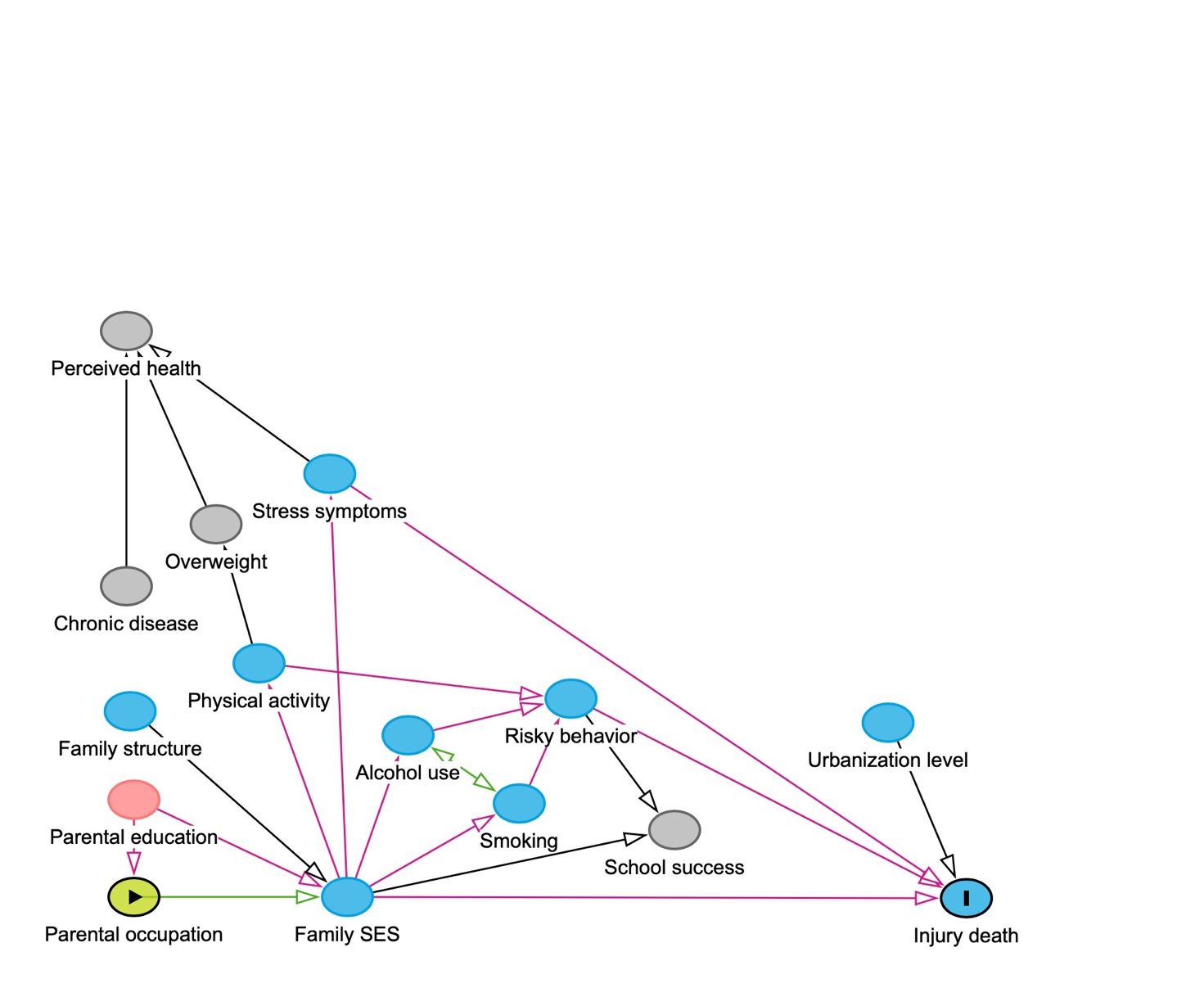
**

**Supplementary figure 11.** DAG:Parental occupational status and the risk of injury death.

Supplement: Supplementary file 15 — Supplementary Material 15. Supplementary Fig. 11. DAG: Parental occupational status and the risk of injury death. [file 12889_2025_23214_MOESM15_ESM.docx]

**
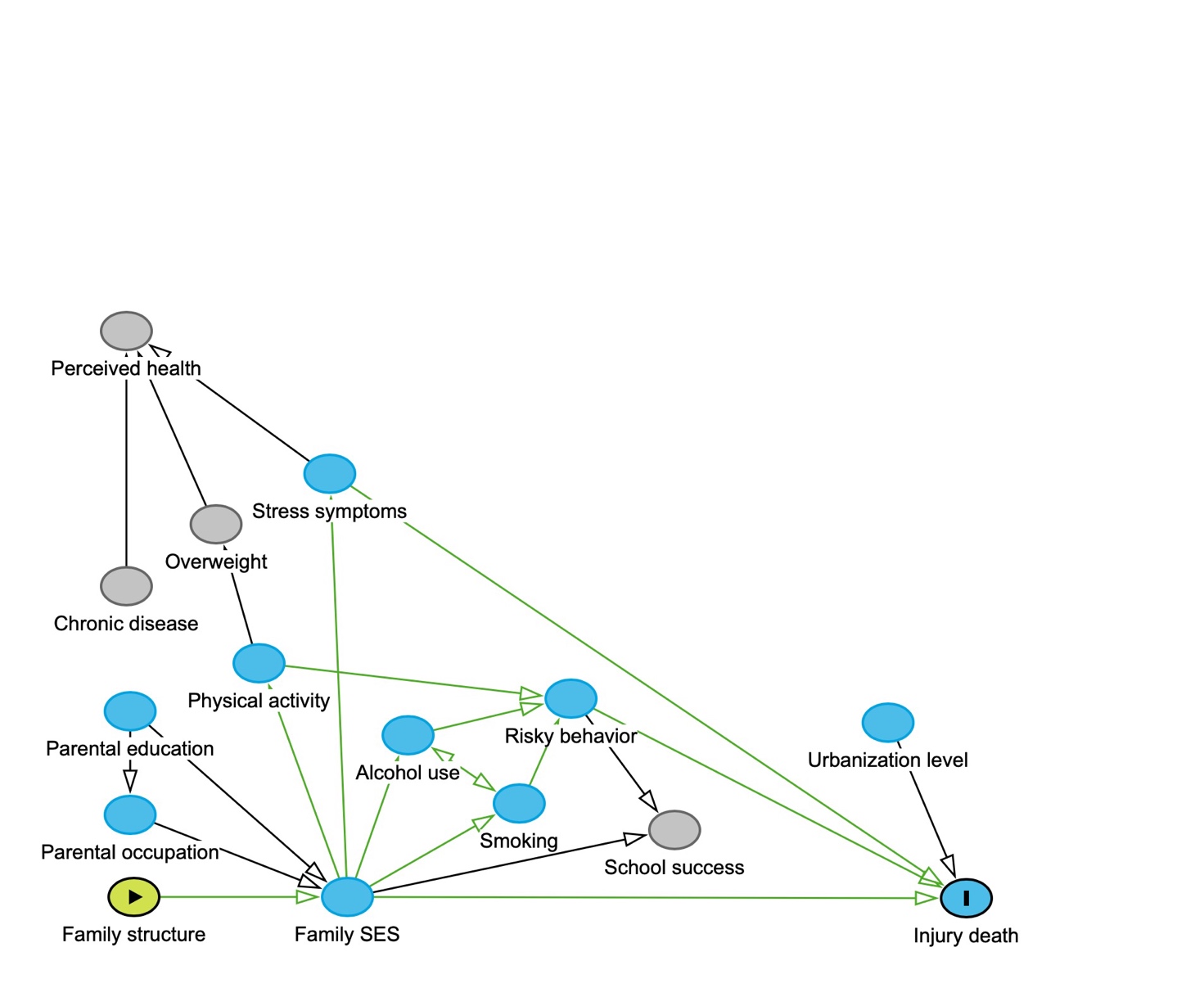
**

**Supplementary figure 12.** DAG:Family structure and the risk of injury death.

Supplement: Supplementary file 16 — Supplementary Material 16. Supplementary Fig. 12. DAG: Family structure and the risk of injury death. [file 12889_2025_23214_MOESM16_ESM.docx]

**
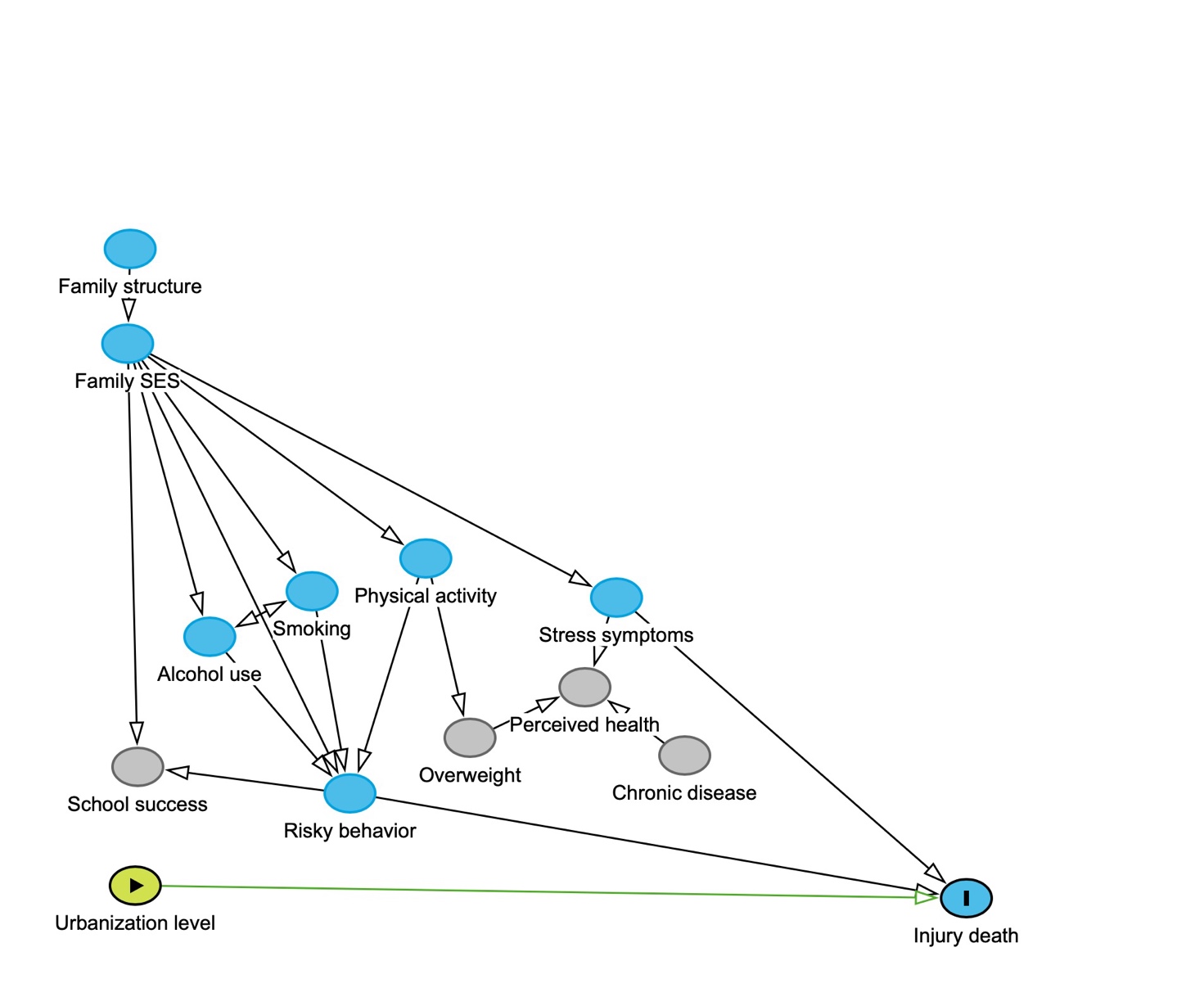
Supplementary figure 13** DAG:Urbanization level of residence and the risk of injury death.

Supplement: Supplementary file 17 — Supplementary Material 17. Supplementary Fig. 13 DAG: Urbanization level of residence and the risk of injury death. [file 12889_2025_23214_MOESM17_ESM.docx]
